# Genetically Predicted Higher Educational Attainment Decreases the Risk of COVID-19 Susceptibility and Severity: A Mendelian Randomization Study

**DOI:** 10.3389/fpubh.2021.731962

**Published:** 2021-12-23

**Authors:** Zhongyu Jian, Menghua Wang, Xi Jin, Xin Wei

**Affiliations:** ^1^Department of Urology, Institute of Urology (Laboratory of Reconstructive Urology), West China Hospital, Sichuan University, Chengdu, China; ^2^West China Biomedical Big Data Center, Sichuan University, Chengdu, China

**Keywords:** COVID-19, Mendelian randomization, susceptibility, severity, educational attainment

## Abstract

**Background:** Prior observational studies indicated that lower educational attainment (EA) is associated with higher COVID-19 risk, while these findings were vulnerable to bias from confounding factors. We aimed to clarify the causal effect of EA on COVID-19 susceptibility, hospitalization, and severity using Mendelian randomization (MR).

**Methods:** We identified genetic instruments for EA from a large genome-wide association study (GWAS) (*n* = 1,131,881). Summary statistics for COVID-19 susceptibility (112,612 cases and 2,474,079 controls), hospitalization (24,274 cases and 2,061,529 controls), and severity (8,779 cases and 1,001,875 controls) were obtained from the COVID-19 Host Genetics Initiative. We used the single-variable MR (SVMR) and the multivariable MR (MVMR) controlling intelligence, income, body mass index, vigorous physical activity, sedentary behavior, smoking, and alcohol consumption to estimate the total and direct effects of EA on COVID-19 outcomes. Inverse variance weighted was the primary analysis method. All the statistical analyses were performed using R software.

**Results:** Results from the SVMR showed that genetically predicted higher EA was correlated with a lower risk of COVID-19 susceptibility [odds ratio (OR) 0.86, 95% CI 0.84–0.89], hospitalization (OR 0.67, 95% CI 0.62–0.73), and severity (OR 0.67, 95% CI 0.58–0.79). EA still maintained its effects in most of the MVMR.

**Conclusion:** Educational attainment is a predictor for susceptibility, hospitalization, and severity of COVID-19 disease. Population with lower EA should be provided with a higher prioritization to public health resources to decrease the morbidity and mortality of COVID-19.

## Introduction

COVID-19 first emerged in December 2019 and has become a worldwide pandemic currently (1). At the time of April 18, 2021, there have been 140,821,384 confirmed cases and 3,013,042 deaths globally (2). In this condition, it is essential to identify high-risk groups that need special attention (3). Particularly, with the available but limited supply of COVID-19 vaccines, a crucial challenge is prioritizing groups to receive vaccines (4–6). There have been discussions about vaccine prioritization for racial minorities (4) and diabetes groups (7), since prior studies report these groups are more vulnerable to COVID-19 disease. In contrast, more evidence for other potential high-risk groups such as the population with low educational attainment (EA) is needed.

Educational attainment is a well-established social determinant of health (8) and correlates with many diseases (9–11). Prior observational studies indicated that the same might have happened during the current COVID-19 pandemic and a population with lower EA was found at a higher risk of susceptibility, hospitalization, and mortality of COVID-19 (12–14). However, conventional observational studies lacking randomization designs are generally prone to confounding factors (15). Randomized controlled trials, on the other hand, cannot be conducted.

Mendelian randomization (MR) is a method that uses genetic variants correlated with an exposure (such as EA) to evaluate whether it has a causal effect on the disease outcome (such as COVID-19 susceptibility) (16), which is less likely to be influenced by unmeasured confounding than observational studies (17). MR is especially useful for exploring causal pathways when the risk factors are difficult to randomize (18). There have been several MR studies exploring the risk factors for COVID-19 (18–20).

An extension of the single-variable MR (SVMR) is the multivariable MR (MVMR), which can incorporate genetic variants associated with several exposures into the same model (21). Since EA was identified to correlate with intelligence, income, body mass index (BMI), vigorous physical activity, sedentary behavior, smoking, and alcohol consumption in a prior study (22), we would also perform the paired MVMR to investigate the direct effects of EA when controlling these exposures separately and explore whether the effects of EA on COVID-19 were independent of them.

Therefore, in this study, we aimed to evaluate the total and direct effects of EA on the susceptibility, hospitalization, and severity of COVID-19 using the SVMR and the MVMR separately, trying to provide evidence for public health resources allocation and targeting prevention planning.

## Materials and Methods

### Genome-Wide Association Study (GWAS) Data Sources for EA

We extracted single-nucleotide polymorphisms (SNPs) correlated with EA from a published GWAS meta-analysis, which included 71 studies with 1,131,881 European-descent individuals in total (23) and this is the largest GWAS of EA to date. We used SNPs at the genome-wide significance of *p* < 5 × 10^−8^ and excluded those in potential linkage disequilibrium (*r*^2^ > 0.01), being palindromic with intermediate allele frequencies or not reported in COVID-19 outcome GWAS datasets. SNP coefficients were expressed in SD units (SD = 4.2 years). We used the *F* statistics to evaluate the strength of genetic variants. One prior study using the similar SNPs with ours reported a median *F* statistics of 45 (24), indicating that the validity of genetic variants was generally reliable. Proportion of variance explained by included SNPs was calculated according to one prior study (25). We presented a detailed description of EA in Supplementary Table 1. Due to privacy policy, we only used summary statistics excluding 23andMe in our MVMR.

### Genome-Wide Association Study Data Sources for Other Related Resources

Summary statistics for intelligence (26), income, BMI (27), vigorous physical activity (28), sedentary behavior, smoking (29), and alcohol consumption (29) were obtained. We placed the detailed information about these exposures in Supplementary Table 1.

### Genome-Wide Association Study Data Sources for COVID-19 Outcomes

We obtained GWAS data for COVID-19 outcomes from the 6th round of the COVID-19 Host Genetics Initiative (COVID-19 HGI), which was conducted on mixed ancestry and released at June 15, 2021. Detailed information about the COVID-19 HGI has been described elsewhere (30). Three different phenotypes, including susceptibility (112,612 cases and 2,474,079 controls), hospitalization (24,274 cases and 2,061,529 controls), and severity (8,779 cases and 1,001,875 controls), were analyzed in our MR analyses. We placed the definition and sample size of each phenotype in Supplementary Table 2.

Additionally, we would also use the 5th round summary data for European ancestry only to conducted sensitivity analyses.

### Statistical Power

Genetic instrumental SNPs explained 3.4% of the variance for EA (Supplementary Table 3). We used an online tool to calculate the power to detect the casual estimates (31) https://sb452.shinyapps.io/power/. With a type I error of 5%, we have sufficient statistical power to detect the difference in the risk of susceptibility, hospitalization, and severity of COVID-19 using all the cohorts. When using the instrumental SNPs after excluding those from 23andMe, similar results were observed. We presented detailed information in Supplementary Table 3.

### Statistical Analysis

In the SVMR, we used the random-effects inverse-variance weighted (IVW) method to estimate the total effects of EA on COVID-19 susceptibility, hospitalization, and severity separately. To validate the results of this study, several sensitivity analyses, including MR-Egger, weighted median, and weighted mode, were conducted additionally. MR-Egger is a method, which can detect and adjust for directional pleiotropy (32). Weighted median method allows up to half of the genetic variants to be invalid (33). As for the weighted mode, it is robust to horizontal pleiotropy (34). If we obtained similar results from all these four MR models, our findings would be more robust. We also used the Cochran's *Q* test to detect possible heterogeneity across individual SNPs and intercept from MR-Egger regression to detect directional pleiotropy.

Next, we used overlapping SNPs as instruments. We applied the random-effects IVW framework to estimate the direct effects of EA in the MVMR analyses after controlling intelligence, income, BMI, vigorous physical activity, sedentary behavior, smoking, and alcohol consumption separately. Although there was partial overlap among GWAS data and we did not have individual-level data, we still calculated the conditional F-statistic approximately for reference. The calculated results were presented in Supplementary Table 4.

Genome-wide association study data could be accessed through the MR-Base platform (35, 36). All the statistical analyses were conducted using R software.

## Results

### Genetically Predicted EA on COVID-19 Susceptibility

**Figure 1:**
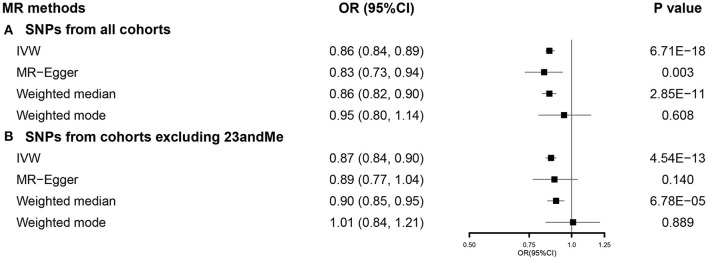
The SVMR forest plot of EA on COVID-19 susceptibility using SNPs from **(A)** all the cohorts and **(B)** all the cohorts excluding 23andMe. COVID-19, coronavirus disease 2019; MR, Mendelian randomization; OR, odds ratio; SNPs, single-nucleotide polymorphisms; IVW, inverse-variance weighted; SVMR, single-variable MR; EA, educational attainment.

After selection, we used 751 SNPs for our SVMR analysis investigating the total effect of EA on COVID-19 susceptibility (Supplementary Figure 1). Primary analysis using IVW indicated that a 1-SD increase in EA was related to a lower risk of COVID-19 susceptibility [odds ratio (OR) 0.86, 95% CI 0.84–0.89]. This effect was consistent across MR-Egger and weighted median, while no significant relationship was observed in weighted mode (Figure 1A). No directional pleiotropy was found (*p* = 0.485), while potential heterogeneity was detected. When using 462 SNPs after excluding those from 23andMe (Supplementary Figure 2), we observed similar results (Figure 1B). Detailed information was presented in Supplementary Table 5. Results from sensitivity analyses also supported our findings (Supplementary Figure 3).

**Figure 2:**
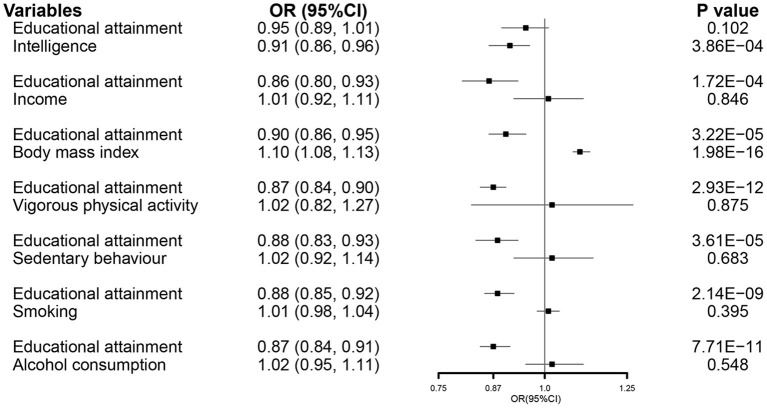
The MVMR forest plot of EA on COVID-19 susceptibility controlling intelligence, income, body mass index, vigorous physical activity, sedentary behavior, smoking, and alcohol consumption separately. COVID-19, coronavirus disease 2019; OR, odds ratio; MVMR, multivariable Mendelian randomization; EA, educational attainment.

In the MVMR analysis, EA retained its association with susceptibility of COVID-19 after controlling all other exposures except for intelligence (Figure 2).

### Genetically Predicted EA on COVID-19 Hospitalization

**Figure 3:**
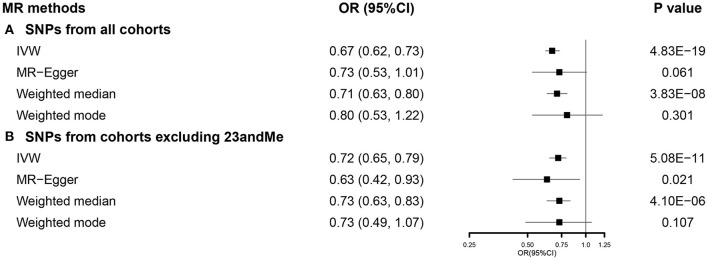
The SVMR forest plot of EA on COVID-19 hospitalization using SNPs from **(A)** all the cohorts and **(B)** all the cohorts excluding 23andMe. COVID-19, coronavirus disease 2019; MR, Mendelian randomization; OR, odds ratio; SNPs, single-nucleotide polymorphisms; IVW, inverse-variance weighted; SVMR, single-variable MR; EA, educational attainment.

We used 751 SNPs in our SVMR analysis investigating the total effect of EA on COVID-19 hospitalization (Supplementary Figure 1). Primary analysis using IVW indicated that a 1-SD increase in EA was correlated with a lower risk of COVID-19 hospitalization (OR 0.67, 95% CI 0.62–0.73). This association was consistent with weighted median, while no causal relationship was observed in MR-Egger and weighted mode (Figure 3A). Results from the MR-Egger intercept and heterogeneity test were presented in Supplementary Table 5. When using 462 SNPs after excluding those from 23andMe (Supplementary Figure 2), we observed similar results (Figure 3B). Detailed results were presented in Supplementary Table 5. Results from sensitivity analyses also supported our findings (Supplementary Figure 3).

**Figure 4:**
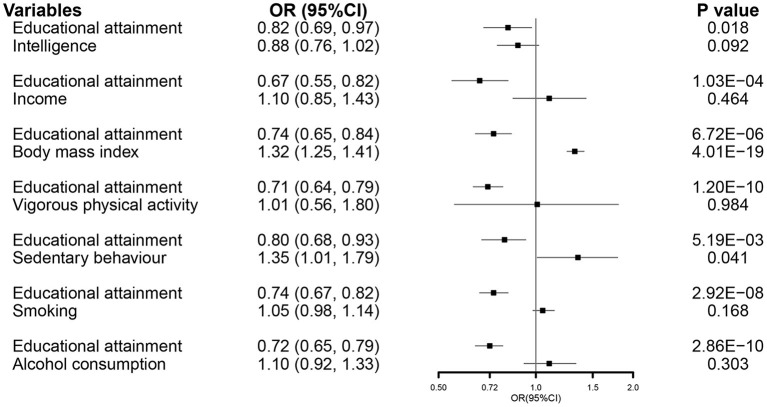
The MVMR forest plot of EA on COVID-19 hospitalization controlling intelligence, income, body mass index, vigorous physical activity, sedentary behavior, smoking, and alcohol consumption separately. COVID-19, coronavirus disease 2019; OR, odds ratio; MVMR, multivariable Mendelian randomization; EA, educational attainment.

In the MVMR analysis, EA maintained its association with susceptibility of COVID-19 after accounting for all other exposures (Figure 4).

### Genetically Predicted EA on COVID-19 Severity

**Figure 5:**
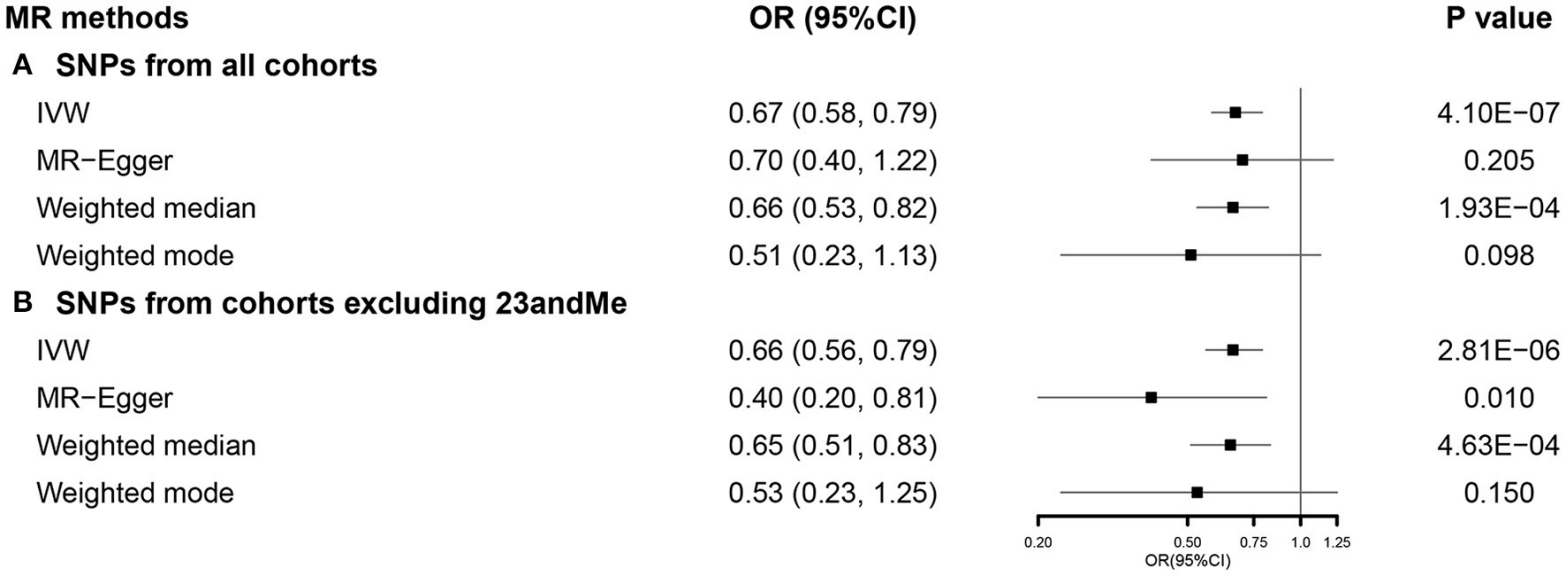
The SVMR forest plot of EA on COVID-19 severity using SNPs from **(A)** all the cohorts and **(B)** all the cohorts excluding 23andMe. COVID-19, coronavirus disease 2019; MR, Mendelian randomization; OR, odds ratio; SNPs, single-nucleotide polymorphisms; IVW, inverse-variance weighted; SVMR, single-variable MR; EA, educational attainment.

After selection, we used 744 SNPs in our SVMR analysis investigating the total effect of EA on COVID-19 severity (Supplementary Figure 1). Primary analysis using IVW indicated that a 1-SD increase in EA was correlated with a lower COVID-19 severity (OR 0.67, 95% CI 0.58–0.79). This association was consistent with weighted median, while no causal relationship was observed in MR-Egger and weighted mode (Figure 5A). Results from the MR-Egger intercept and heterogeneity test were presented in Supplementary Table 5. When using 455 SNPs after excluding those from 23andMe (Supplementary Figure 2), the effect of EA on COVID-19 severity was observed among all the methods, except weighted mode (Figure 5B). Results from sensitivity analyses also supported our findings (Supplementary Figure 3).

**Figure 6:**
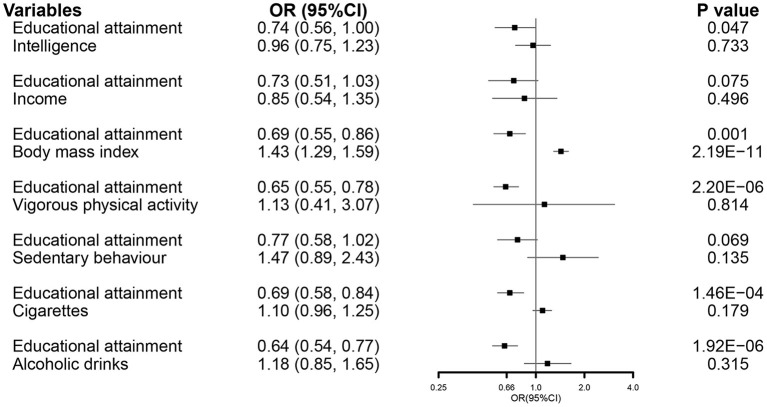
The MVMR forest plot of EA on COVID-19 severity controlling intelligence, income, body mass index, vigorous physical activity, sedentary behavior, smoking, and alcohol consumption separately. COVID-19, coronavirus disease 2019; OR, odds ratio; MVMR, multivariable Mendelian randomization; EA, educational attainment.

In the MVMR analysis, EA maintained a direct effect on the severity of COVID-19 after controlling all other related exposures, except income and sedentary behavior (Figure 6).

## Discussion

In this study, using both the SVMR and MVMR, we found genetically higher EA was related to a lower risk of susceptibility, hospitalization, and severity of COVID-19.

Using data from UK Biobank, a prior study reported that lower EA was correlated with a higher COVID-19 infection risk [relative risk (RR) 2.00, 95% CI 1.66–2.42] (13), indicating EA might be a possible predictor for COVID-19 infection. However, the sample size of this study was relatively small, with only 948 cases enrolled, making their results less reliable. While in this study, using the largest GWAS of EA and latest summary statistics for COVID-19, we have sufficient statistical power and found that EA is correlated with COVID-19 susceptibility. The underlying mechanism between EA and COVID-19 susceptibility could be partly explained by frontline jobs usually without a requirement of an advanced degree (37), thus individuals with lower EA being more likely to be infected. Second, people with lower EA tend to have a lower socioeconomic status. As a result, they are more likely to live with multiple close generations and, therefore, at greater risk of contracting COVID-19 (4).

Hospitalization is another important COVID-19 outcome and the population with lower EA was reported at a higher rate of hospitalization in a prior observational study (38). However, a major disadvantage of this study is that they only used data from 5 New York City boroughs and their sample might not be representative enough. While in this study, this shortcoming was overcome and we found that EA was a predictor for COVID-19 hospitalization in both the SVMR and MVMR. In addition to COVID-19, EA has been identified as a risk factor for hospitalization among many infectious diseases such as pneumonia and bacteremia (39, 40). Data from a Danish population-based case–control study indicated that the population with a short duration of education had a substantially higher risk of bacteremia than those with long duration (39). The underlying mechanism between lower EA and higher risk of hospitalization might be mediated by overcrowding, poor housing conditions, and hygienic practices.

In addition, this study also found that EA was a predictor for COVID-19 severity, which was in accordance with one prior MR study from Japan (41). However, comparing with the study from Japan, we applied the latest 6th round of COVID-19 summary statistics in this study with a larger sample. Several hypotheses are elucidating the mechanism between EA and COVID-19 severity. First, individuals with lower EA and socioeconomic positions are more likely to be affected by job stress such as unemployment, which might lead to a higher risk of immune system disruption and comorbidities (42). So far, both the weak immunity and the presence of comorbidities are recognized risk factors of COVID-19 severity (43, 44). Besides, population with lower EA is more likely to have unhealthy behaviors such as smoking and an unbalanced diet (45), while these unhealthy behaviors have been recognized as risk factors of COVID-19 mortality or severity (46, 47). Additionally, lower EA generally correlates with lower income and socioeconomic status, which means that they have limited access to healthcare (48) and once they are infected, they might not get treatment in time and turn to be critically ill or even dead.

As for the MVMR, we found that EA still maintained its effects on COVID-19 outcomes under almost all the conditions. This showed that the effects of EA were generally independent of these exposures. One limitation of our MVMR was that part of the conditional F-statistic was relatively low. However, since there was partial overlap, the conditional F-statistic might not be accurate enough and should be interpreted cautiously (21).

This study has several strengths. First, using the largest GWAS of EA with 1.1 million participants and the latest summary statistics for COVID-19 from the 6th round of the COVID-19 HGI, we have enough statistical power. Second, although MR study is still prone to potential sources of bias, it is generally less vulnerable to confounding factors than observational studies. Third, in addition to the SVMR, we also conducted the MVMR analyses and results showed that the effects of EA on COVID-19 outcomes were generally independent of some other exposures. However, this study could not be devoid of limitations. First, since individuals with no or mild symptoms are less likely to test for COVID-19 and there might be potential selection bias in this study. Second, COVID-19 outcome ascertainment methods were not exactly the same among all the cohorts in the COVID-19 HGI, leading to the potential heterogeneity. However, we were unable to estimate how these differences might influence our findings since we only had access to the overall summary statistics. Third, in order to increase the sample size and statistical power, we used the overall COVID-19 HGI GWAS in our primary analyses, which was conducted on mixed ancestry and might lead to some potential population stratification biases. However, we have conducted sensitivity analyses using the summary statistics for European only. Fourth, one prior study reported that using GWAS of education-related traits might be biased from population stratification (49). Another limitation of our MR is the partial overlap of participants (e.g., UKB) in the exposure and outcome datasets, which might lead to possible bias (50). However, bias caused by sample overlap would likely to be minimal for both the continuous and binary outcomes (50) and it has also been shown that 2-sample MR methods may be safely used in single sample provided the data are derived from large biobanks (51), as is the case in our analysis. Last, EA is a complex phenotype and might correlate with some cofounders. Although we observed no directional pleiotropy and the MVMR analyses also showed that the effects of EA on COVID-19 outcomes were generally independent of some other exposures, it is still possible that confounding and pleiotropy may be present.

## Conclusion

Our MR analyses indicated that EA is a predictor for susceptibility, hospitalization, and severity of COVID-19 disease. Population with lower EA should be provided with a higher prioritization of public health resources to decrease the morbidity and mortality of COVID-19.

## Data Availability Statement

Publicly available datasets were analyzed in this study and can be accessed via the references we used.

## Ethics Statement

Ethical review and approval was not required for the study on human participants in accordance with the local legislation and institutional requirements. Written informed consent for participation was not required for this study in accordance with the national legislation and the institutional requirements.

## Author Contributions

ZJ, MW, XJ, and XW: designing the study. ZJ and MW: carrying out the study, analyzing the data, and writing the article. XJ and XW: revising the article. All authors read and approved the final manuscript.

## Funding

This study was funded by Post-Doctor Research Project, West China Hospital, Sichuan University (2020HXBH016).

## Conflict of Interest

The authors declare that the research was conducted in the absence of any commercial or financial relationships that could be construed as a potential conflict of interest.

## Publisher's Note

All claims expressed in this article are solely those of the authors and do not necessarily represent those of their affiliated organizations, or those of the publisher, the editors and the reviewers. Any product that may be evaluated in this article, or claim that may be made by its manufacturer, is not guaranteed or endorsed by the publisher.
