# Supplementary material for: Genetically Predicted Higher Educational Attainment Decreases the Risk of COVID-19 Susceptibility and Severity: A Mendelian Randomization Study
Source: Front Public Health. 2021 Dec 23;9:731962. doi: 10.3389/fpubh.2021.731962 (PMC8732991; doi:10.3389/fpubh.2021.731962)
Supplement: Supplementary file 1 [file Data_Sheet_1.docx]

Supplementary Material

**Supplementary Table 1.** Genetic instrument sources and description

| Exposures | Type | Definition | Units | Sample size | Population | Consortium | MR base ID | Notes: N UKB | Notes: PMID |
| --- | --- | --- | --- | --- | --- | --- | --- | --- | --- |
| EA | Continuous | Educational years measured at an age of at least 30 | SD in years | 1,131,881 | European | SSGAC | NA^#^ | 442,183 | 30038396 |
| EA | Continuous | Educational years measured at an age of at least 30 | SD in years | 766,345 | European | SSGAC | ieu-a-1239 | 442,183 | 30038396 |
| Intelligence | Continuous | Operationalized to index a common latent g factor underlying multiple dimensions of cognitive functioning | SD in intelligence scores | 269,867 | European | CTGlab | ebi-a-GCST006250 | 195,653 | 29942086 |
| Income | Categorical Ordered | Average total household income before tax | SD in pounds | 397,751 | European | MRC-IEU | ukb-b-7408 | 397,751 | Pre-publication |
| BMI | Continuous | Body mass divided by the square of the body height | kg/m^2^ | 681,275 | European | GIANT | ieu-b-40 | 456,426 | 30124842 |
| Vigorous physical activity | Categorical Ordered | 10 minutes or more of vigorous physical activity (These are activities that make you sweat or breathe hard such as fast cycling, aerobics, heavy lifting) | logOR | 261,055 | European | NA | ebi-a-GCST006098 | 261,055 | 29899525 |
| Sedentary behavior | Categorical Ordered | Hours of television viewing per day | SD in hours/day | 437,887 | European | MRC-IEU | ukb-b-5192 | 437,887 | Pre-publication |
| Smoking | Continuous | Average number of cigarettes smoked per day, either as a current smoker or former smoker, and whether self-rolled or manufactured are smoked | Cigarettes/day | 337,334 | European | GSCAN | ieu-b-25 | 120,744 | 30643251 |
| Alcohol consumption | Continuous | Average number of drinks a participant reported drinking each week, aggregated across all types of alcohol | Alcoholic drinks/week | 335,394 | European | GSCAN | ieu-b-73 | NA | 30643251 |

MR: Mendelian randomization; UKB: UK Biobank; EA: Educational attainment; SD, standard deviation; SSGAC: Social Science Genetic Association Consortium; CTGlab: Complex Trait Genetics; MRC-IEU: Medical Research Center-Integrative Epidemiology Center (UK Bristol); BMI: body mass index; GIANT: Genetic Investigation of ANthropometric Traits; GSCAN: GWAS & Sequencing Consortium of Alcohol and Nicotine Use

^#^ Summary statistics including participants from 23andMe are not available

**Supplementary Table 2.** Phenotype source and description

| Outcomes | Definition | Sample size (N case/ N control) | Consortium | Notes: N UKB | Notes: PMID |
| --- | --- | --- | --- | --- | --- |
| COVID-19 Susceptibility | Self-reported, EHR/ICD coding/ Physician OR laboratory confirmed SARS-CoV-2 infection | 2,586,691 (112,612/2,474,079) | COVID-19 Host Genetics Initiative | 433,715 | 32404885 |
| COVID-19 Hospitalization | Hospitalized laboratory confirmed SARS-CoV-2 infection, hospitalization due to corona-related symptoms | 2,085,803 (24,274/2,061,529) | COVID-19 Host Genetics Initiative | 436,043 | 32404885 |
| COVID-19 Severity | Death OR respiratory support AND hospitalization with COVID19 as primary reason for admission. | 1,010,654 (8,779/1,001,875) | COVID-19 Host Genetics Initiative | 420,531 | 32404885 |

UKB, UK Biobank

**Supplementary Table 3. Statistical power to detect difference in susceptibility, hospitalization, and severity of COVID-19**

| Exposure (EA) data sources | Proportion of variance explained | Outcomes | Outcome Sample size | Type-I error rate | Proportion of cases | Calculated power when setting the causal effects at 0.8 |
| --- | --- | --- | --- | --- | --- | --- |
| All cohort | 3.4% | COVID-19 Susceptibility | 2,586,691 | 0.05 | 4.4% | 100% |
|  | 3.4% | COVID-19 Hospitalization | 2,085,803 | 0.05 | 1.2% | 100% |
|  | 3.4% | COVID-19 Severity | 1,010,654 | 0.05 | 0.9% | 97% |
| All cohort excluding 23andMe | 2.7% | COVID-19 Susceptibility | 2,586,691 | 0.05 | 4.4% | 100% |
|  | 2.7% | COVID-19 Hospitalization | 2,085,803 | 0.05 | 1.2% | 100% |
|  | 2.7% | COVID-19 Severity | 1,010,654 | 0.05 | 0.9% | 92.8% |

EA: Educational attainment

**Supplementary Table 4.** Conditional F-statistics of educational attainment for instrument strength in MVMR

|  | COVID-19 Susceptibility | COVID-19 Hospitalization | COVID-19 Severity |
| --- | --- | --- | --- |
| MVMR with Intelligence | 6.6 | 6.6 | 6.7 |
| MVMR with Income | 2.7 | 2.7 | 2.8 |
| MVMR with Body mass index | 10.1 | 10.1 | 10.1 |
| MVMR with Vigorous physical activity | 25.4 | 25.4 | 29.0 |
| MVMR with Sedentary behaviour | 6.1 | 6.1 | 6.2 |
| MVMR with Smoking | 32.5 | 32.5 | 32.0 |
| MVMR with Alcohol consumption | 42.7 | 42.7 | 42.8 |

MVMR, multivariable Mendelian randomization

**Supplementary Table 5.** Summary on SVMR results of EA on COVID-19 susceptibility, hospitalization, and severity

| **EXPOSURE** | **OUTCOME** | **MR RESULTS** | | | | | **HETEROGENEITY** | | **PLEIOTROPY** | |
| --- | --- | --- | --- | --- | --- | --- | --- | --- | --- | --- |
|  |  | **Methods** | **N SNPs** | **OR** | **95%CI** | ***P* value** | **METHODS** | ***P* value** | **METHODS** | ***P* value** |
| EA ^a^ | COVID-19 Susceptibility | IVW | 751 | 0.86 | 0.84-0.89 | 6.71E-18 | IVW | 7.13E-05 |  |  |
|  |  | MR-Egger | 751 | 0.83 | 0.73-0.94 | 0.003 | MR-Egger | 6.80E-05 | MR-Egger intercept | 0.485 |
|  |  | Weighted median | 751 | 0.86 | 0.82-0.90 | 2.85E-11 |  |  |  |  |
|  |  | Weighted mode | 751 | 0.95 | 0.80-1.14 | 0.608 |  |  |  |  |
|  |  |  |  |  |  |  |  |  |  |  |
| EA ^b^ | COVID-19 Susceptibility | IVW | 462 | 0.87 | 0.84-0.90 | 4.54E-13 | IVW | <0.001 |  |  |
|  |  | MR-Egger | 462 | 0.89 | 0.77-1.04 | 0.140 | MR-Egger | <0.001 | MR-Egger intercept | 0.723 |
|  |  | Weighted median | 462 | 0.90 | 0.85-0.95 | 6.78E-05 |  |  |  |  |
|  |  | Weighted mode | 462 | 1.01 | 0.84-1.21 | 0.889 |  |  |  |  |
|  |  |  |  |  |  |  |  |  |  |  |
| EA ^a^ | COVID-19 Hospitalization | IVW | 751 | 0.67 | 0.62-0.73 | 4.83E-19 | IVW | 5.16E-05 |  |  |
|  |  | MR-Egger | 751 | 0.73 | 0.53-1.01 | 0.061 | MR-Egger | 4.82E-05 | MR-Egger intercept | 0.574 |
|  |  | Weighted median | 751 | 0.71 | 0.63-0.80 | 3.83E-08 |  |  |  |  |
|  |  | Weighted mode | 751 | 0.80 | 0.53-1.22 | 0.301 |  |  |  |  |
|  |  |  |  |  |  |  |  |  |  |  |
| EA ^b^ | COVID-19 Hospitalization | IVW | 462 | 0.72 | 0.65-0.79 | 5.08E-11 | IVW | 5.22E-05 |  |  |
|  |  | MR-Egger | 462 | 0.63 | 0.42-0.93 | 0.021 | MR-Egger | 4.92E-05 | MR-Egger intercept | 0.489 |
|  |  | Weighted median | 462 | 0.73 | 0.63-0.83 | 4.10E-06 |  |  |  |  |
|  |  | Weighted mode | 462 | 0.73 | 0.49-1.07 | 0.107 |  |  |  |  |
|  |  |  |  |  |  |  |  |  |  |  |
| EA ^a^ | COVID-19 Severity | IVW | 744 | 0.67 | 0.58-0.79 | 4.10E-07 | IVW | 0.001 |  |  |
|  |  | MR-Egger | 744 | 0.70 | 0.40-1.22 | 0.205 | MR-Egger | <0.001 | MR-Egger intercept | 0.908 |
|  |  | Weighted median | 744 | 0.66 | 0.53-0.82 | 1.93E-04 |  |  |  |  |
|  |  | Weighted mode | 744 | 0.51 | 0.23-1.13 | 0.098 |  |  |  |  |
|  |  |  |  |  |  |  |  |  |  |  |
| EA ^b^ | COVID-19 Severity | IVW | 455 | 0.66 | 0.56-0.79 | 2.81E-06 | IVW | <0.001 |  |  |
|  |  | MR-Egger | 455 | 0.40 | 0.20-0.81 | 0.010 | MR-Egger | <0.001 | MR-Egger intercept | 0.148 |
|  |  | Weighted median | 455 | 0.65 | 0.51-0.83 | 4.63E-04 |  |  |  |  |
|  |  | Weighted mode | 455 | 0.53 | 0.23-1.25 | 0.150 |  |  |  |  |

SVMR, single-variable Mendelian randomization; EA, educational attainment; SNP, single-nucleotide polymorphism; OR, odds ratio; CI, confidence interval; IVW, inverse-variance weighted

**^a^** From all cohorts

^b^ From all cohorts excluding 23andMe


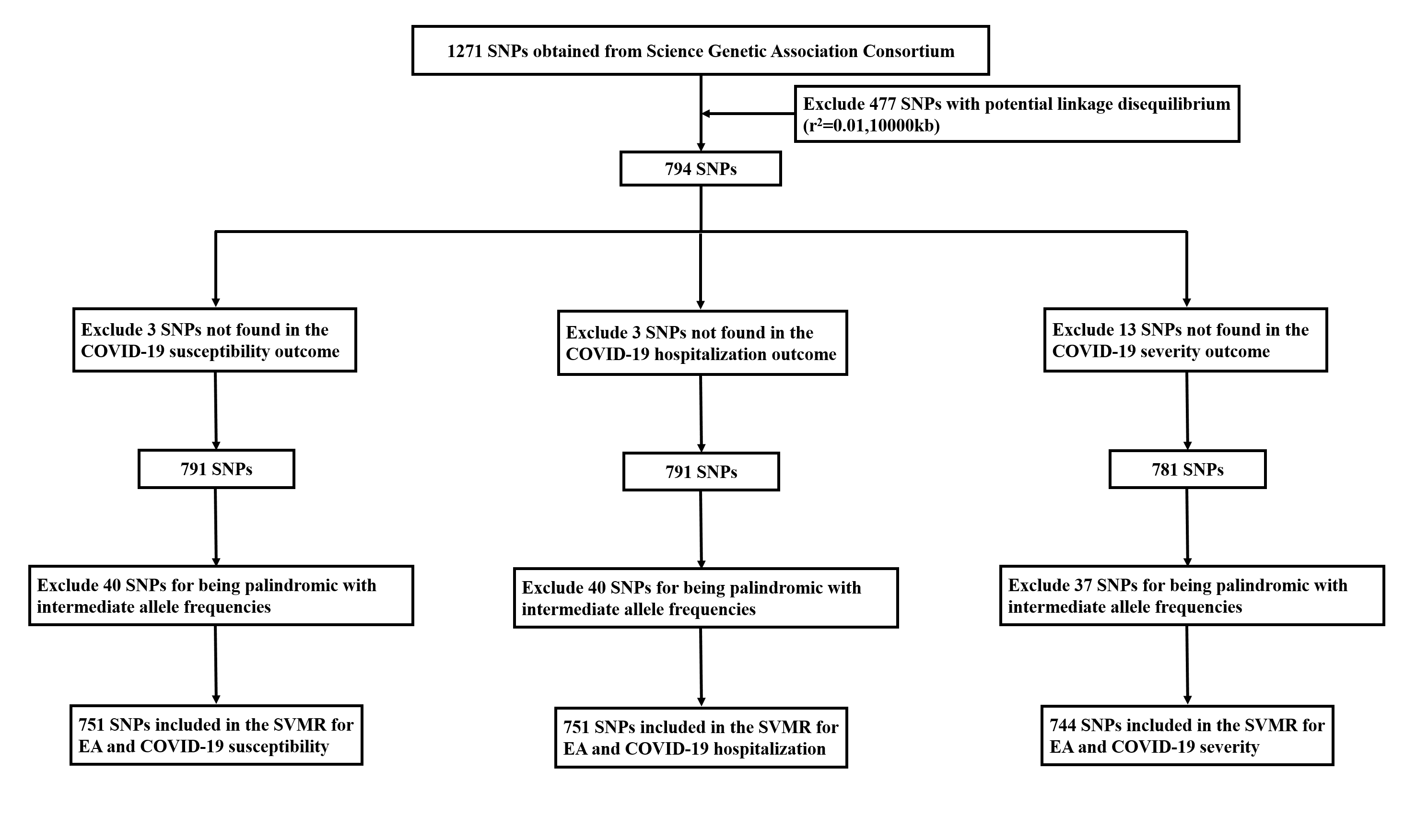


**Supplementary Figure 1.** The flowchart of SNPs selection for SVMR to evaluate the total effect of EA on COVID-19 susceptibility, hospitalization, and severity separately. SNP, single-nucleotide polymorphism; SVMR, Single-variable Mendelian randomization; EA, educational attainment


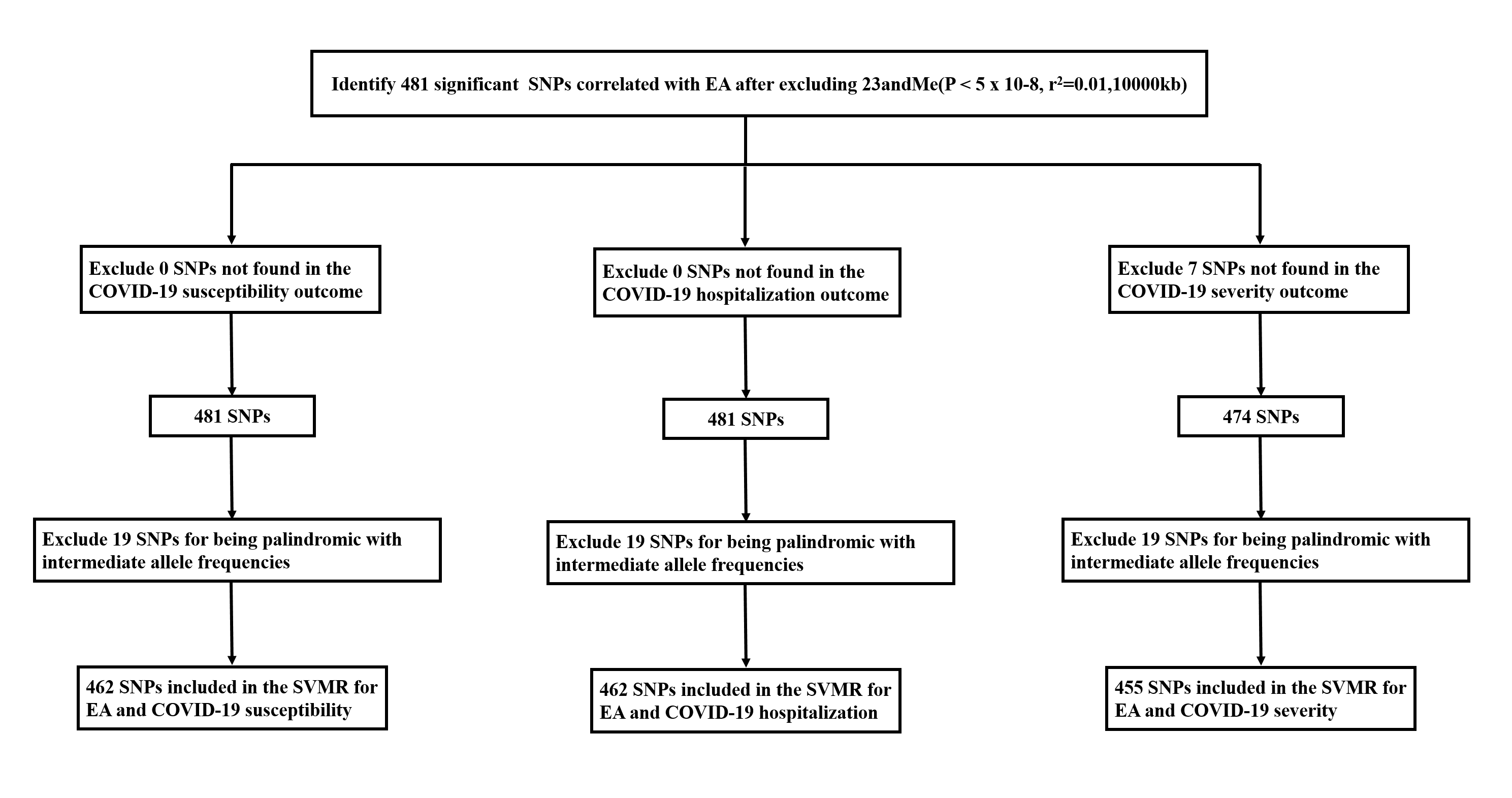


**Supplementary Figure 2.** The flowchart of SNPs selection after excluding 23andMe for SVMR to evaluate the total effect of EA on COVID-19 susceptibility, hospitalization, and severity separately. SNP, single-nucleotide polymorphism; SVMR, Single-variable Mendelian randomization; EA, educational attainment


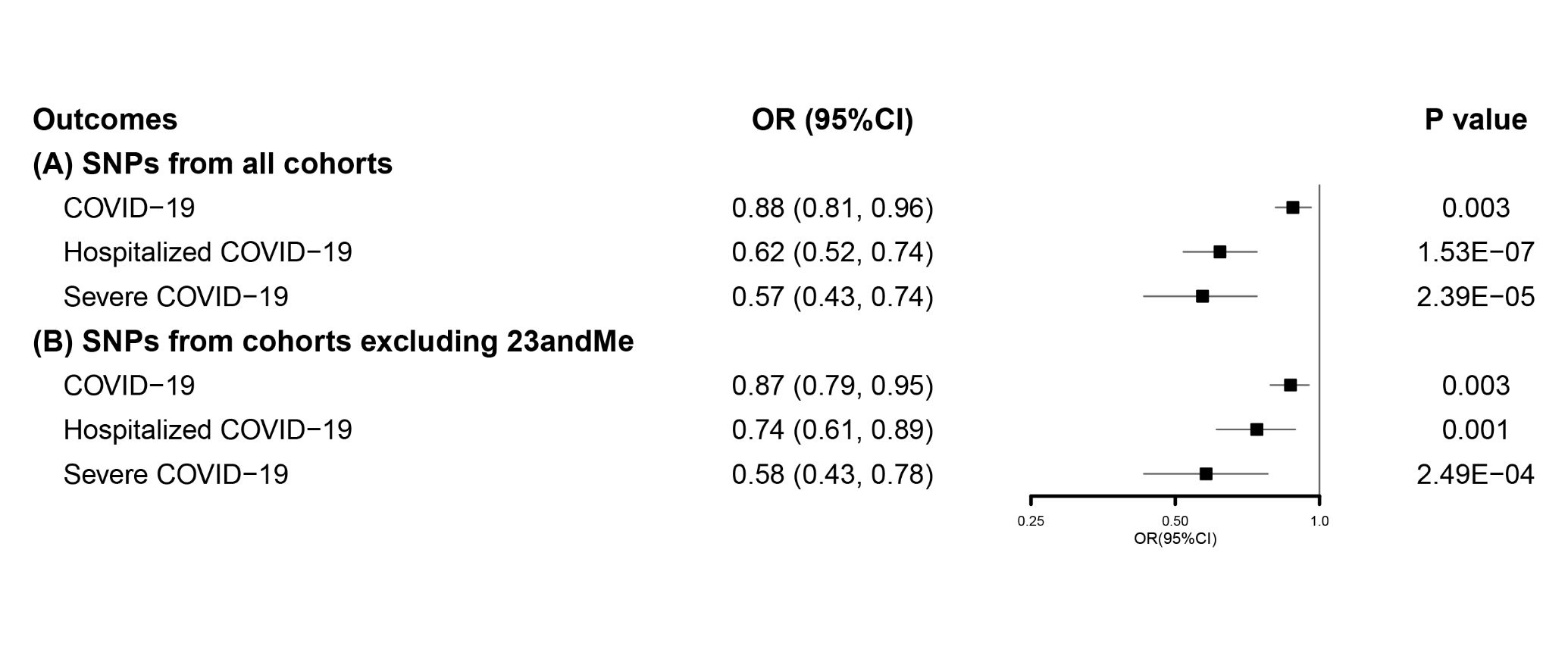
**Supplementary Figure 3.**  Sensitivity analyses of SVMR using the summary statistics for European ancestry from the fifth round of COVID-19 Host Genetics Initiative. SVMR, Single-variable Mendelian randomization
